# The Vacation

**Published:** 1861-07

**Authors:** 


### MEDICAL CRITIC

AND

### PSYCHOLOGICAL

### JOURNAL.

JULY, 1861.

### Art. I.—THE VACATION.

The season for holiday-making is fast approaching. The polite and the political world, the professional and the mercantile, are already beginning to reckon the weeks or days before they can start once more by steamboat or by rail, and leave us poor scribblers far, far behind them, to the summer dust and solitude of London.*

The joy of travelling is universal. To many it is new life, for at heart we are all of us children, or we ought to be so—"pleased with a feather, tickled with a straw." When the morning is bright and clear, and the day for starting has arrived, how cheerfully do we bid a short farewell to home, and hasten on board the steamer, the bustle and confusion of which only give additional zest to the momentary sense of pleasure. The breeze blows afresh as the boat quits the harbour, heaves upon the coming waves, and cuts through the crested foam; health enters by the eye; the optic nerve is engaged with new sights; the invalid forgets himself, and time flies without his knowing it.

To the jaded Londoner, whose nerves are jarred by the incessant railroad, change of air and scene is almost indispensable.

* "I reluctantly left Paris, and hastily drove through the summer dust and solitude of London."—*Gibbon's Autobiography.* What he regretted in 1765 we heartily respond to in 1861. But if Gibbon was delighted with the capital of France a hundred years ago, what would he say to it now? For though the Paris of his day no longer exists, yet most of its social attractions still remain, and the modern city has risen, like the phœnix, young, bright, and new, from the ashes of revolution, bloodshed, disaster, and dynastic change.

At least once a year every one who possibly can, leaves the great metropolis. Fifty years ago, only the privileged sons of fortune could venture on travelling; now, every one travels. Fifty years ago, those strange mental and bodily ailments usually ascribed to medullary softening or exhaustion, were only exceptional cases; but now, almost every case partakes somewhat of their character: the wan eye, the languid gait, the prone and bloodless hand, emphatically bespeak their origin. Odd feelings of numbness, without loss of motor power, an undefined foreboding of impending evil, puny remorse or capricious regrets, irresolution, and disgust of life, indicate either some deep-seated mischief of the nervous centre, or the effects of a moral shock, that has penetrated to the very seat and citadel of life. Intensity is the order of the day; velocity is the soul of business; decision is a matter of necessity; and yet, in fact, the world is only what it ever was. No one can comprehend at a single glance the precise bearing of circumstances and events which speed away from sight as quickly as they had come into view; they are come and gone while one is looking on. It is too late to decide except upon the spot. Decide we must, whether the decision be right or wrong; and much must be left to time, and chance, and the chapter of accidents. Earnest but feeble minds find the task too much for them, and they shrink unconsciously from the weight of a responsibility which is forced upon them without the delay proper for deliberation. Their nerves are overstretched, and they totter or fall.

The master minds are so few, that we may consider them as the giants looked up to and implicitly followed by the crowd. The rest of mankind are nothing more than ordinary mortals. Nevertheless, we are all of us hurried away, both giants and dwarfs, by a gigantic order of affairs, to which there is nothing analogous in the course of history. In spite of ourselves, we are all *fast* men. Every one suffers from it. Health is no longer what it was. Nervous maladies take the lead, fevers are of a low type. Formerly the latter were acute and inflammatory, now they are just the reverse. The treatment that was successful then, would it be successful now? Would not the present plan of sustaining the failing powers of life have in those days only added fuel to fire? The whole is changed—the mode of living, the features of disease, and the style of practice; all is changed, and so are we ourselves. Everything tends to promote a direct pressure on the nervous system, to raise a call for incessant excitement. There is no interval of rest. The great town roars from morning till night, and from night till morning. A short hour or so before dawn, its granite pavement may be silent; but at sunrise, or before sunrise in winter, the hum begins again, goes on increasing, and shortly rises into one long continued roar. To sleep, is it possible? The intellect, does it ever sleep? Dreams, are they not the waking thoughts, hopes, fears, and ambition or despair of the day? And what is the consequence? This patient has lost the use of his arm, another is blind or paraplegic, a third is losing his memory and self-possession, and is obliged to resign a lucrative post. They all look ill, and, in fact, are very ill. They all show signs of wasted blood and damaged nutrition of the brain. They are all of them temperate. They none of them smoke, or drink, or do worse. On the contrary, they are upright to a nicety, only the burden of life is too much for them.

The weariness of life, incidental to mental and bodily exhaustion, is a form of disease of peculiar inveteracy. Sometimes it amounts to unmitigated apathy, pervading every function of the frame. To cease to be, is the only desire left, connected with a vague and indefinite idea of leaving life for the purpose of shaking off an incubus weighing heavily on the mind. The nerves are unstrung and no longer respond to the touch of interest or affection. Perhaps, it may be said, that overwrought luxury or care is but a protracted suicide, long-drawn out. Which is the better or the worse, the profligate who destroys himself piecemeal, or the greedy usurer who supplies his needs? What is the value of a great reputation at the bar or in medicine, if you are found dead in your bed, with a distraint in your house, or you win golden opinions from all sorts of people at the cost of more than fifty per cent. on your income? What a life! Late dinners and late hours, rich repasts or meagre meals, hot assemblies, theatres, and saloons, the close and ill-ventilated bedroom, the use of tobacco, and the absolute necessity of having recourse to wine, spirits, or opium as a solace or support, beneath a vast canopy of smoke, that obscures the air and adjacent country for many a mile in circumference; such is the climax of a mode of life as diametrically opposite to health and happiness as light is to darkness, or earth to heaven!

The laws of nature are never transgressed with impunity. In the physical being of man there is no redeeming power analogous to that of repentance in the supernatural order of faith. Damaged functions are seldom or never restored to their primitive integrity. Their further deterioration may be arrested, but the point at which degeneration halts remains permanently fixed below the normal standard of health. Nought is left but a broken constitution.

But we are wandering from our point. We have forgotten ourselves. Our brown study was caused by the sight of that sickly-looking stranger who has just stepped on board the steamer, and seems pining for fresh air and relaxation from the toils of office. Where is he going? to the Alps or the Pyrenees? It matters not which, for either will do. No scenery is so exhilarating to an enervated patient as that of the mountains. It is particularly renovating to the worn-out Cit. Everything looks enchanting to him. He can scarcely believe his senses. The sight of those distant giant peaks coquetting with the wreaths of clouds around their lofty summits—their wonderful variations of light and shade, and perspective and form—the valleys between and among them scattered over with villages, farms, fields, streams, cattle, hedges, woods, and pathways, looking so small, clear, and distinct in the depths below; or the sombre pine forests, such as those so ably depicted by Gaspar Poussin, clothing the heights above, below, and around him, vanishing beneath his feet, over his head, or far away, as far as the eye can reach, here, there, and everywhere—unfold a moving diorama or dissolving view that annihilates time and space, and transports the mind in fancy to the land of dreams.

Lofty elevations give rise to singular feelings in the breasts of those unused to them. There is something preternatural in their effect. But there is more than the sensation of novelty and grandeur which they inspire. There is a material alteration of the whole frame. Heights such as those of Mexico, Peru, Bolivia, and the awful Himalayas, produce phenomena, both in animals and men, of the gravest kind. It is styled the Alpine climate, and its effects are everywhere the same. Saussure, on the top of Mont Blanc; Humboldt and Boussingault on the peaks of Teneriffe or Chimborazo; or Moorcroft on the terrible altitudes of the Hindoo Coosh; each and all experienced precisely the same feelings at the same elevations. The diminished amount of oxygen, or the greatly reduced barometrical pressure, or the free electricity so clearly demonstrated by the frequency and severity of thunderstorms, hurried the circulation and breathing, and made the arteries of the head pump with unusual energy. Bodily exertion did not cause it, for those who rode on horseback, or were carried in cars or palanquins, suffered the same. Humboldt's guides bled from the nose, mouth, and eyes—a sanguineous exudation rather than a direct hæmorrhage. Aëronauts suffer in the same way, and so do divers into great depths of the ocean: the mean surface of the earth being the only level adapted to the functions of life. The digestive organs are sympathetically affected. There is a sensation of fulness at the pit of the stomach; loss of appetite, nausea, or vomiting. The thirst is sometimes excessive—a desire for cold drinks, and a positive aversion to wine or spirits, although cordials are by no means prejudicial; indeed, it is astonishing what a large quantity of ardent spirits can be consumed at these great heights without intoxication. Every traveller mentions this curious fact.

At extreme elevations muscular strength nearly ceases. Beasts of burden sink under their loads and lose their strength much more rapidly than man. They stagger, stumble, fall down, and often die from sheer exhaustion, as their whitened bones abundantly testify along the passes of the Himalayas or the Andes. But the most important effect is that produced directly on the nervous system, signified by giddiness, drowsiness, headache, singing in the ears, and an almost incontrollable propensity to lie down and sleep. These symptoms occur at the extremest heights. At lower elevations they manifest themselves in a lower degree. Still lower, they give place to a sense of lightness and buoyancy, as if the ground were elastic, and the feet seem to skim along the surface as if flying. The traveller feels himself so braced up and alert, that he can walk farther and more easily than ever he could do on the plains he has just ascended from. Life is sensibly invigorated. There is a kind of intoxication of delight. Hence the benefit to valetudinarians. A prolonged sojourn on the heights takes away all these good effects, and at length does harm. The tongue is parched, the eye bloodshot, the cheeks pale, with a congested red spot in their centre, and the skin ceases to perspire. Very sensitive persons experience these unpleasant sensations at an elevation of five thousand feet above the sea-level, particularly if riding instead of walking. Exercise on foot in general soon restores the circulation.

Animals are affected in the same manner as man, but not in the same degree. When the Spaniards went to Bolivia, they took their cattle along with them, and they found, to their utter astonishment, that their bulls had lost their ferocity and pugnacity. The torédors could scarcely stir up their rage, or, if they did, the bulls fell down at the first encounter or turned tail, to the infinite disgust of the spectators assembled to witness their favourite amusement. Possibly the animals may have become acclimatized at last, for Boussingault witnessed a regular bull-fight at Quito, which is some thousand feet lower than Paz in Bolivia, where the animals first failed. Cats die at 12,000 feet above the sea, from tetanic spasms. Dogs live longer than cats, particularly if born in the locality, but they are often subject to fits, like those of pups. Hares and rabbits live at a great elevation, but the race soon ceases. Poultry quickly die. Horses and mules become acclimatized, but they require the greatest care, and must be allowed to halt frequently in the course of a journey. The Lamas of Peru and the Yaks of Mongolia suffer like the mules and horses. Bears and wolves exist beneath the snow-line. Eagles and condors, those denizens of air, live at an elevation of 20,000 feet above the sea-level. Butterflies, spiders, and house-flies were observed by Saussure at 12,000 feet, and by Bonpland still higher than that. The chamois, the gazelle, and the izzard, enjoy a free and independent existence among the highest crags and precipices, but then it is their native place, and they are, as it were, to the manner born.

In Europe none of these bodily sufferings are so strongly marked as they are in Asia and America. Those who have ascended Mont Blanc, or, like MM. Desor and Agassiz, passed a day and a night on the glaciers of Aars, have not remained long enough in those inhospitable regions to enable us to form a fair estimate of their injurious effects. It is from travellers who have dwelt for some time on the Himalayas or the Andes that we learn what is the real pathology of those elevated localities. Acute inflammatory fevers stand at the top of the list. Dyspnœa is a very common complaint. It is a kind of asthma, produced by a dry, cold, and rarefied atmosphere. Animals suffer from it as well as man. Natives of the plains, the corpulent, and robust, suffer the most. It is the worst in dry seasons. Among new comers, the digestion, which is at first much distressed, recovers quickly enough, but the dyspnœa and lassitude consequent upon it continue for many months, and in some cases are never cured, except by returning to the lowest levels. The complaint is seldom fatal. There may be apoplexy or pulmonary congestion in those so disposed, but in general it is an inconvenience rather than a disease. The maladies peculiar to hot climates are unknown in the mountains, and ague is cured by a residence among them.*

It is evident that the changes effected within the animal economy by a short residence in these elevated regions are of the most important kind. It cannot fail to be most favourable to the worn-out inhabitant of great cities. The stimulating influence that it exerts on the nervous system in particular is the very remedy that his case requires. The respiration, the circulation, and the process of digestion, are all of them powerfully acted on and promoted. In enervated and wasted constitutions, these beneficial results are the most obvious. But, chief of all, is the good that it accomplishes on the mental faculties. What is most needed, and the least likely to be obtained, in the treatment of mental maladies, not only at home, but also in the same place, and in the midst of kindred associations, is a complete and sudden diversion of thought and feeling. There is nothing within reach so long as the patient is confined to the circle of his

---

* These remarks are taken from *Les Climats des Montagnes*, par Dr. Lombard. Genève. 1858.

own country, relations, and habitual pursuits. They must be all changed. New scenes, new thoughts, new ways, and new food, are the *primum mobile* of cure. Nowhere can they be found in so ample a degree as among the solitude and grandeur of a mountain pass—such as that of the Via Mala, in the Grisons; or the Port de Venasque, at the entrance into Spain, in the Pyrenees, near Luchon. We appeal to the *initiated*, and ask whether such points of sight can ever become vulgarized or lose their beneficial effects by long acquaintance? They are too unique ever to become commonplace or be forgotten. Like the ocean, always the same yet always new, they fix the eye in wonder, contemplation, and repose; and the novice is satisfied with a feeling of the sublime that is never effaced.

The mind shakes off the phantoms of the past, recovers its tone and manliness, and life is renewed. A rich background is furnished to the drudgery and business of the world. The number of ideas is multiplied. There is no end to the stores of nature. Who would ever have supposed that these solid peaks have a language of their own? and yet the *voices of the mountains* are as old as the days of Strabo; and Humboldt himself uses the expression, which he has borrowed from the ancient geographer, where he is describing the Lipari islands and Phlegræan fields of old.*

An anonymous writer in the *Edinburgh Philosophical Journal*, "On Peculiar Noises heard in Particular Districts," mentions a dull moaning sound that came from the Maladetta in the Pyrenees at a moment when the sky was cloudless, and *Mont Maudit* was distinctly visible in the transparent atmosphere of the south. In his *Views of Nature*, Humboldt mentions the organ-like sounds heard at sunrise on the banks of the Orinoco, proceeding apparently from the granite rocks that had been overheated by the intense rays of the sun the day before.† Mr. Scrope, in his excellent work on extinct volcanoes, states that some rocks absorb moisture, and give it out again with a hissing sound, and a considerable disengagement of air bubbles. The rock called domite is one of these, of which the Puy de Dôme is nothing more than a huge mass. It is a pumice, the product of ignition, and looks grey and naked enough as it towers above the dingy, antiquated town of Clermont-Ferrand, in Auvergne.‡ The clinkstone and trachyte of Mont Mezen, Haute Loire, as well as of Mont Dore, one of the range of Puys, might readily yield a distant reverberation

---

* *Strabo*, lib. vi. 276. Humboldt, *Kosmos*, v. p. 263.

† The sound proceeding from the Memnonian Statue in the sands of Ancient Egypt might be thus accounted for.

‡ Scrope's *Extinct Volcanoes*, p. 46. Murray. 1858.

from the constant effect of meteoric agencies, detrition, and decay. Mr. Weld, in his recent work on the *Pyrenees*, likewise alludes to the mysterious sounds wandering through the solitudes of the mountains, and answering each other in echoes from height to height. Perhaps these noises may be accounted for by currents of air sweeping round the lofty peaks on high, while a profound calm reigns at their base.* Around the sugar-loaf point of Mont Ventoux, 8000 feet high, in the neighbourhood of Avignon, fleecy clouds may be seen circulating on the calmest day.

But let us descend. The back wheels are locked; the horses are put on the full trot; the drivers shout and smack their whips; the road turns sharp round; the vehicle swings, you overhang the depths below, and a moment afterwards you are driving down the next slope in the opposite direction, to run the same risk at the next sharp turning of the steeply-inclined plane. This desperate experiment is repeated many times before you reach the long-looked-for bottom. The slightest accident to the vehicle, an inadvertence of the driver's, the tripping of a horse, would inevitably send the whole freight over the brink, several thousand feet deep. Had we experienced the same embarrassment on the heights as we did in the valley, our fate would have been sealed. We had just crossed a shallow mountain torrent, and were rumbling along the grassy plain beside it, when the seventh horse, or leader, ridden by a lad *en postillon*, began jibbing, backed on its three fellows behind, and they again backed on the three wheelers, which threw the vehicle awry out of the roadway. One of the conductors alighted, ran to the leader's head, re-adjusted something wrong in the harness, and everything was quickly put to rights; but the same occurrence on the heights would have toppled bag, baggage, horses, and all, headlong over the precipice, and then you would never have heard from your ill-fated contributor again.

---

* *The Pyrenees, West and East.* By C. R. Weld. p. 194. Longman. 1859.

